# Beyond the Ivory Tower: Perception of academic global surgery by surgeons in low- and middle-income countries

**DOI:** 10.1371/journal.pgph.0002979

**Published:** 2024-03-14

**Authors:** Arinzechukwu Nwagbata, Rohini Dutta, Anusha Jayaram, Neil Thivalapill, Samarvir Jain, Isabella Faria, Isaac G. Alty, Anita Gadgil, Nobhojit Roy, Nakul P. Raykar, Global Surgery Survey Team

**Affiliations:** 1 Harvard Medical School, Boston, Massachusetts, United States of America; 2 Department of Trauma, Burn, and Critical Care, Brigham and Women’s Hospital, Boston, Massachusetts, United States of America; 3 Program in Global Surgery and Social Change, Department of Global Health and Social Medicine, Harvard Medical School, Boston, Massachusetts, United States of America; 4 WHO Collaborating Centre for Research in Surgical Care Delivery in Low-Middle Income Countries, Mumbai, India; 5 Department of Surgery, Beth Israel Deaconess Medical Center, Boston, Massachusetts, United States of America; 6 Feinberg School of Medicine, Northwestern University, Chicago, Illinois, United States of America; 7 The George Institute for Global Health, New Delhi, India; 8 Karolinska Institutet, Stockholm, Sweden; Royal Infirmary of Edinburgh, UNITED KINGDOM

## Abstract

Interest in global surgery has surged amongst academics and practitioners in high-income countries (HICs), but it is unclear how frontline surgical practitioners in low-resource environments perceive the new field or its benefit. Our objective was to assess perceptions of academic global surgery amongst surgeons in low- and middle-income countries (LMICs). We conducted a cross-sectional e-survey among surgical trainees and consultants in 62 LMICs, as defined by the World Bank in 2020. This paper is a sub-analysis highlighting the perception of academic surgery and the association between practice setting and responses using Pearson’s Chi-square test. Analyses were completed using Stata15. The survey received 416 responses, including 173 consultants (41.6%), 221 residents (53.1%), 8 medical graduates (1.9%), and 14 fellows (3.4%). Of these, 72 responses (17.3%) were from low-income countries, 137 (32.9%) from lower-middle-income countries, and 207 (49.8%) from upper-middle-income countries. 286 respondents (68.8%) practiced in urban areas, 34 (8.2%) in rural areas, and 84 (20.2%) in both rural and urban areas. Only 185 (44.58%) were familiar with the term “global surgery.” However, 326 (79.3%) agreed that collaborating with HIC surgeons for research is beneficial to being a global surgeon, 323 (78.8%) agreed that having an HIC co-author improves likelihood of publication in a reputable journal, 337 (81.6%) agreed that securing research funding is difficult in their country, 195 (47.3%) agreed that their institutions consider research for promotion, 252 (61.0%) agreed that they can combine research and clinical practice, and 336 (82%) are willing to train HIC medical students and residents. A majority of these LMIC surgeons noted limited academic incentives to perform research in the field. The academic global surgery community should take note and foster equitable collaborations to ensure that this critical segment of stakeholders is engaged and has fewer barriers to participation.

## Introduction

Academic global surgery (AGS) seeks to improve surgical conditions affecting vulnerable populations, often in resource-poor environments where health access may be limited [1]. It involves the integration of clinical outcomes and basic, translational, and health services research into the practice of global surgery [2]. With the establishment of the Lancet Commission on Global Surgery in the last decade, academic surgeons in high-income countries (HICs) have increased partnerships with surgeons in low- and middle-income countries (LMICs) [3]. These partnerships have progressively depended on reciprocal clinical, research, and educational collaborations.

Many academic global surgeons in both HICs and LMICs rely on ensuing scholarly writings and responsibilities from these research engagements in addition to participation in surgical professional organizations for academic advancement [4]. While partnerships with frontline faculty in LMICs have supported the academic careers of HIC academic global surgeons, these perks have not always been reciprocated [5]. Such an outcome is due to both institutional pressures and discrepant perceptions between HIC volunteers and local hosts [6]. Studies characterizing unidirectional relationships in global surgery have reported these relationships to be neocolonial with a focus on the HIC institution’s agenda [6–10].

Understanding the perception of AGS among surgeons in LMICs is thus vital to characterizing their acknowledgment of these current practices in global surgery, as well as their involvement, challenges, and willingness to broaden their responsibilities in their roles as drivers of global surgery. This involves gaining insight into their valuation of the evolving dynamics of the HIC partnership which is expanding to include bidirectional training for surgical trainees and international funding opportunities for research and development in global surgery [2, 11].

Our study aimed to explore the perception of AGS and its benefits among surgery, anesthesia, and obstetric care practitioners and trainees from multiple LMICs, while also evaluating perceived institutional support for surgeons in LMICs. The results of our study contribute to a deeper understanding of the local dynamics of academic global surgery, with the potential to drive changes that improve surgical care for vulnerable populations.

## Materials and methods

### Study design, population, and setting

We analyzed the perception towards AGS from a cross-sectional e-survey of surgical residents, fellows, and consultants/specialists in LMICs as defined by the World Bank in 2020 [12]. Eligible participants were workers in health facilities (private or government) in the departments of general surgery, surgical subspecialties (neurosurgery, vascular surgery, plastic surgery, trauma surgery, cardiothoracic surgery, urology, surgical oncology, pediatric surgery, otolaryngology surgery, ophthalmologic surgery, orthopedic surgery), obstetrics and gynecology, or anesthesia. Eligibility was irrespective of previous global surgery background and experience.

### Survey design

We conducted pilot studies with 40 surgical, anesthetic, and obstetric care providers in LMICs to test the survey for language and comprehension. We pragmatically chose data variables that were objective, easily standardized, and relevant, to minimize missing data and maximize data quality. The survey incorporated adaptive questioning with each page containing approximately 7 to 8 questionnaire items over five pages. We also utilized branching logic based on participant categories.

### Data collection

We collected data using an anonymous, self-administered online survey on a secure, password-encrypted Research Electronic Data Capture (REDCap) from 1st February 2022 to 21st March 2022. To spearhead the recruitment process, we enlisted 73 country leads representing 62 LMICs, and in some instances, assigned more than one lead per country before distributing the survey. The country leads utilized a standardized email template and letter prepared by the steering committee of the project team to contact medical schools, hospitals, and professional societies, inviting them to participate in and disseminate the survey. During the study period, we sent these invitations to 89 medical schools, surgical societies, and organizations via email. Our team collaborated with 23 organizations and medical schools across 13 countries to support survey dissemination. Participation in the survey was voluntary, and we encouraged participants to share the survey with their colleagues.

### Data analysis

We conducted our analysis using Stata 15 (StataCorp) [13]. We compared data between low-income countries (LICs), lower-middle-income countries (LMCs), and upper-middle-income countries (UMICs) as defined by the World Bank in 2020. We started by using descriptive statistics, employing one-way ANOVA to measure the mean differences among groups. For categorical variables, we utilized the Pearson chi-square test to compare the three groups. A significance level of 0.05 was applied to all tests. To uphold data integrity, we conducted a data cleaning process on the anonymous survey. We identified and removed incomplete and duplicate entries through a systematic approach. This involved leveraging REDCap-generated participant Record IDs to eliminate redundancy, ensuring a more accurate and reliable dataset.

### Ethical approval

The study received exemption of Ethical approval from the Institutional Review Board of Mass General Brigham (2021P002088). Every participant in the study formally consented on the first page of the REDCAP survey, where the informed consent section outlined the study’s purpose and stressed the voluntary nature of participation. Participants who did not provide consent were unable to proceed to the subsequent pages of the survey.

## Results

**Fig 1:**
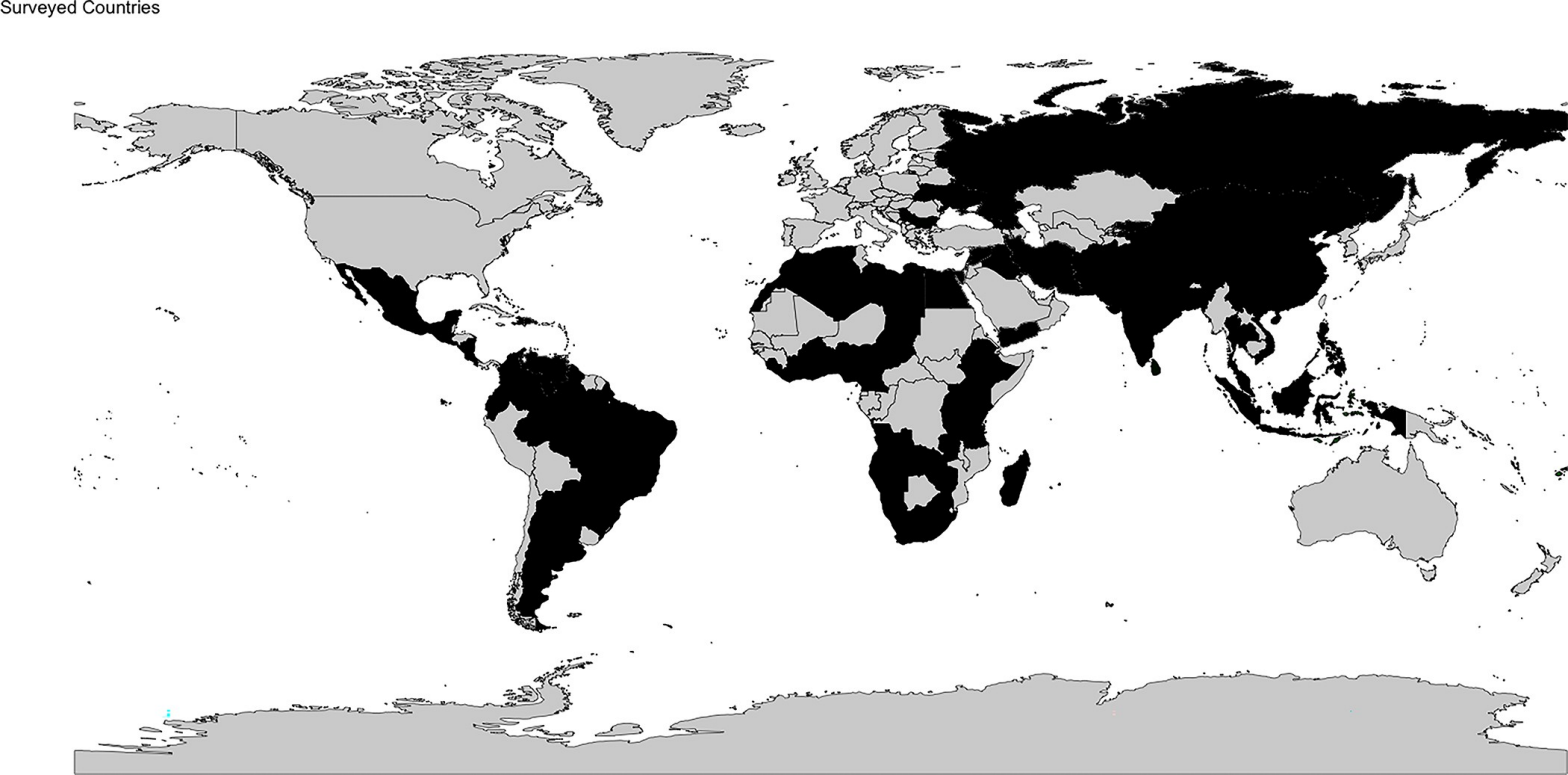
Visual representation of the countries where survey respondents are located. Made with Natural Earth. Free vector and raster map data @ naturalearthdata.com.

We received 416 responses, with a median respondent age of 34.7 years (Table 1). Of these respondents, 173 were consultants/attendings (41.6%) and 243 were residents and fellows (58.4%). A majority of respondents (207, 49.8%) were from UMICs, while 137 (32.9%) were from LMCs and 72 (17.3%) from LICs (Fig 1).

**Table 1:** Survey respondent demographic and professional characteristics.

	Total N = 416	LIC* N = 72	LMC* N = 137	UMIC* N = 207	p-value
**Age**	34.7 (23–70)	32.5 (29.5–39.5)	34.0 (29.0–39.0)	29.0 (27.0–37.5)	<0.001
**Gender**					0.104
Man	279 (67.1%)	57 (79.2%)	89 (65.0%)	133 (64.5%)	
Woman	130 (31.3%)	14 (19.4%)	48 (35.0%)	68 (32.9%)	
Non-binary	2 (0.5%)	0 (0.0%)	0 (0.0%)	2 (1.0%)	
Prefer not to answer	5 (1.2%)	1 (1.4%)	0 (0.0%)	4 (1.9%)	
**Level of Medical Training**					0.038
Medical Graduate	8 (1.9%)	1 (1.4%)	3 (2.2%)	4 (1.9%)	
Resident/Registrar/Trainee	221 (53.1%)	31 (43.1%)	65 (47.4%)	125 (60.4%)	
Fellow	14 (3.4%)	1 (1.4%)	8 (5.8%)	5 (2.4%)	
Specialist/Consultant/Attending	173 (41.6%)	39 (54.2%)	61 (44.5%)	73 (35.3%)	
**Specialty)/Specialty of interest (if student)**					< 0.001
General Surgery	218 (52.5%)	27 (37.5%)	71 (52.2%)	120 (58%)	
Anaesthesia/ Critical Care	42 (10.1)	3 (4.2%)	22 (16.2%)	17 (8.2%)	
Obstetrics/Gynecology	45 (10.8)	13 (18.1%)	19 (16.1%)	13 (6.3%)	
Other Surgical Subspecialty	110 (26.5)	29 (40.3%)	24 (17.6%)	57 (27.5%)	
**Setting of Healthcare Practice**					< 0.001
Rural	34 (8.1%)	9 (12.5%)	14 (10.2%)	11 (5.3%)	
Urban	286 (68.8%)	36 (50.0%)	93 (67.9%)	157 (75.8%)	
Both	84 (20.2%)	26 (36.1%)	27 (19.7%)	31 (15.0%)	
N/A—Student	12 (2.9%)	1 (1.4%)	3 (2.2%)	8 (3.9%)	
**Setting Grown Up In**					< 0.001
Rural	54 (13.0%)	17 (23.9%)	18 (13.1%)	19 (9.2%)	
Urban	291 (70.1%)	40 (56.3%)	88 (64.2%)	163 (78.7%)	
Both	70 (16.9%)	14 (19.7%)	31 (22.6%)	25 (12.1%)	
**Experience working or studying in a medical system outside of your own country**					0.12
No	254 (61.4%)	37 (52.1%)	82 (59.9%)	135 (65.5%)	
Yes	160 (38.6%)	34 (47.9%)	55 (40.1%)	71 (34.5%)	

*The World Bank income group 2020 definition of LIC was used, which encompasses all countries whose gross national income (GNI) per capita is less than US$1,085. LMC encompasses all countries whose GNI per capita is between US$1,085 and US$4,255. UMIC encompasses all countries whose GNI per capita is between US$4,256 and $13,205.

A total of 54 respondents (13.0%) reported practicing in a rural setting, 291 in a non-rural setting (70.1%), and 70 practiced in both settings (16.9%). Less than half of the respondents (160, 38.6%) had experience working or studying in a medical system outside of their country’s medical system. The highest response rate was from practitioners in General Surgery (174, 41.9%), followed by those in Anesthesia/ Intensive/ Critical Care (42, 10.1%). Practitioners in Endocrinologic Surgery (1, 0.2%) and Dental Surgery/OMF (2, 0.5%) had the lowest responses.

In LICs, LMCs, and UMICs, 51 (72.9%), 91 (66.4%), and 110 (53.4%) of respondents, respectively, agreed that they can combine research with clinical practice, but only 195 respondents (47.3% of 416) believe that their institutions consider their research output for promotion (Table 2). Respondents from lower-income countries were more likely to agree that securing funding for research projects is difficult in their countries (91.4% in LICs vs. 86.1% in LMICs vs. 75.2% in UMICs, p = 0.01654). However, there was no significant difference in the number of respondents that agreed that securing funding for research projects is difficult in their institution among the three groups (78.6% in LICs vs. 79.4% in LMCs vs. 73.5% in UMICs, p = 0.3735). Most respondents, 79.3%, believe that collaborating with HIC surgeons in their scholarly works is beneficial, and 82% expressed their willingness to train individuals from HIC institutions in the LMIC setting.

**Table 2:** Univariate analysis of respondents’ perceptions of academic global surgery.

	Total N = 416	LIC N = 72	LMC N = 137	UMIC N = 207	p-value
**I am able to devote time to research with ongoing clinical practice**					0.01115
Agree	252 (61.0%)	51 (72.9%)	91 (66.4%)	110 (53.4%)	
Disagree	110 (26.6%)	10 (14.3%)	31 (22.6%)	69 (33.5%)	
I don’t know/ Unsure	51 (12.3%)	9 (12.9%)	15 (10.9%)	27 (13.1%)	
**My institution takes my research output into consideration for promotion**					0.144
Agree	195 (47.3%)	27 (38.6%)	70 (51.5%)	98 (47.6%)	
Disagree	127 (30.8%)	20 (28.6%)	42 (30.9%)	65 (31.6%)	
I don’t know/ Unsure	90 (21.8%)	23 (32.9%)	24 (17.6%)	43 (20.9%)	
**Securing funding for research projects is difficult in my country**					0.01654
Agree	337 (81.6%)	64 (91.4%)	118 (86.1%)	155 (75.2%)	
Disagree	42 (10.2%)	4 (5.7%)	10 (7.3%)	28 (13.6%)	
I don’t know/ Unsure	34 (8.2%)	2 (2.9%)	9 (6.6%)	23 (11.2%)	
**Securing funding for research projects is difficult in my institution**					0.3735
Agree	313 (76.3%)	55 (78.6%)	108 (79.4%)	150 (73.5%)	
Disagree	57 (13.9%)	6 (8.6%)	17 (12.5%)	34 (16.7%)	
I don’t know/ Unsure	40 (9.8%)	9 (12.9%)	11 (8.1%)	20 (9.8%)	
**In order to be an academic global surgeon, collaborating with HIC surgeons for research projects may be beneficial**					0.1485
Agree	326 (79.3%)	58 (82.9%)	110 (80.3%)	158 (77.5%)	
Disagree	22 (5.4%)	0 (0.0%)	6 (4.4%)	16 (7.8%)	
I don’t know/ Unsure	63 (15.3%)	12 (17.1%)	21 (15.3%)	30 (14.7%)	
**Having a co-author from a HIC will improve the likelihood of publication of my research in a reputable journal**					0.9192
Agree	323 (78.8%)	55 (79.7%)	109 (80.1%)	159 (77.6%)	
Disagree	20 (4.9%)	3 (4.3%)	5 (3.7%)	12 (5.9%)	
I don’t know/ Unsure	67 (16.3%)	11 (15.9%)	22 (16.2%)	34 (16.6%)	
**I am willing to train medical students/surgical trainees/residents from HIC institutions**					0.1146
Agree	336 (82.0%)	61 (89.7%)	117 (85.4%)	158 (77.1%)	
Disagree	18 (4.4%)	1 (1.5%)	5 (3.6%)	12 (5.9%)	
I don’t know/ Unsure	56 (13.7%)	6 (8.8%)	15 (10.9%)	35 (17.1%)	

## Discussion

This study investigated the perception of academic global surgery (AGS) and its benefits among surgery, anesthesia, and obstetric care workers and trainees from multiple LMICs. Among the 416 LMIC surgeons who participated in the study, 61% reported practicing AGS by integrating research into their clinical practice. However, only 47% believed that their research productivity influenced their career advancement within their institutions. The surveyed surgeons still face challenges in securing research funding and achieving greater research impact, and they expressed a preference for collaboration with HIC researchers to address these issues.

While 4.8 billion people reside in LMICs, where many lack access to safe and affordable surgery, the majority of surgical research is predominantly conducted in HICs, leading to research outcomes that are not always relevant or applicable to LMICs [14]. However, LMICs contribute to only about 4% of surgical research, underscoring the need to identify barriers that hinder the advancement of academic research in these countries [15]. In our study, a majority of respondents (61%) demonstrated the ability to conduct research while fulfilling their clinical responsibilities, highlighting their potential to work towards addressing these needs. Despite facing significant challenges such as overwhelming clinical responsibilities, a shortage of research personnel, insufficient data collection resources, and limited funding, these LMIC surgeons continue to demonstrate a strong interest in pursuing surgical research [16]. However, only 45% of global surgery publications listed on PubMed have authors affiliated with LMIC institutions, indicating a gap between these researchers’ commitment and their representation in scholarly publications [17]. If the global surgery community truly appreciates the contributions of LMIC surgeons in global surgery discourse, it is imperative that we tackle the shared and systemic obstacles they face, to enhance their presence and participation.

Increasing recognition and representation of LMIC academic global surgeons in surgical research may play a vital role in their academic achievements. In countries such as Mexico, Argentina, and South Africa, institutional policies and practices place a higher value on research and development compared to teaching, thereby emphasizing the significance of research for academic advancement [18]. Previous literature has indicated that in various other developing countries, particularly within Asian regions, the importance of publishing output in advancing the careers of academic researchers is relatively less prioritized [19]. Our study showed that 61% of participants from Asian regions (74 out of 121) report that their institutions prioritize their research output in promotion considerations, in contrast to 47% among the total respondents. This highlights that research output is comparatively less prioritized in promotion considerations in many other LMICs outside of Asia. Countries such as Ethiopia, Cameroon, and Sri Lanka have reported challenges with institutional administrations that exhibit centralized, bureaucratic, and hierarchical structures, which often hinder research endeavors [20]. These countries, therefore, do not widely observe the practice of promoting individuals based on meritocracy and surgical research experience [20]. While the criteria for recognition within AGS remains uncertain in high-income countries, efforts are being made in these countries to establish comprehensive guidelines for academic progression in the realm of global surgery [15]. Although LMICs may contemplate adopting comparable directives to enhance the scholarly impact of LMIC surgeons within the AGS domain, it is important to acknowledge that this endeavor will be constrained by the limited availability of funding resources for surgical research in these nations.

Currently, the funding of surgical research in LMICs, whether from national or international agencies and charitable organizations, significantly influences each country’s publication output [19]. Our study showed that LMIC surgeons still face obstacles in obtaining national research funding, with a higher proportion of surgeons in lower-income countries experiencing difficulties compared to their counterparts in middle-income countries. Moreover, funding agencies and charitable organizations often dictate the allocation and utilization of their resources, leading to research topics and methodologies that have minimal impact on basic care provision [6]. For example, a majority of the international funds are primarily directed towards supporting research in elective and specialized procedures, rather than focusing on emergency and basic surgery [21]. Additionally, many HIC institutions also do not acknowledge global surgery as a valid academic discipline [2]. Consequently, this hampers the capacity of motivated HIC surgeons to obtain funding for collaborative projects with counterparts in LMICs working to address fundamental needs. However, the system is gradually evolving. Foreign agencies that provide grants to HIC institutions for research in LMICs are revising their management of research funds. There is a growing trend of awarding joint grants to LMIC institutions, with funds allocated to HIC researchers through external contracts [11]. This approach enables LMIC institutions to direct these grants toward relevant research topics and methodologies [11].

### Exploring partnership between HIC and LMIC surgeons

With the increased partnership between HIC and LMIC surgeons over the past decade, host LMIC institutions express concerns about power imbalances, lack of cultural awareness, and unequal benefits [7]. The global surgery community has persistently advocated for fairness and equality in these partnerships, implementing measures to uphold the significance of the autonomous efforts of LMIC surgeons [22]. While there has been a notable rise in global surgery publications over the last decade, the majority of authors involved in global surgery academia between 1987 and 2017 were associated with LMICs [17]. However, in recently published studies on global surgery, the predominant affiliation of authors is solely with HICs [17]. There is, therefore, a recent expectation within the global health community for the inclusion of LMIC counterparts as first or senior authors, aiming to address the increasing disparity [23]. Yet, a study conducted by Ghani et al. in 2021 examining global health publications from 876 journals revealed that 30% of these publications from LMICs did not have any local author listed [24]. Ravi et al., in the same year, found that 45% of authors of global surgery articles were solely affiliated with LMICs. Among these LMIC authors, 46% were associated with LMCs, 28% with UMICs, and 26% with LICs [17]. Thus, it is unsurprising that over 75% of LMIC surgeons hold the belief that the value of their manuscripts can be influenced by co-authorship with HIC counterparts.

Available data explains these LMIC surgeons’ perceptions of surgical journals’ attitudes toward their independent works. The absence of representatives from LMICs on the editorial boards of many surgical journals results in an inadequate representation of research from LMICs or research led by LMIC authors [25]. This circumstance also likely accounts for the limited overall interest of scholarly journals in the field of global surgery [2]. However, there is a positive shift in the culture, with academic journals such as the *Journal of Surgical Research* and *Surgery* now establishing dedicated categories for global surgery publications [2]. Perhaps the editorial boards of these journals will also expand to accommodate representation from LMICs.

An emerging trend within the collaboration between HICs and LMICs involves HIC global surgery participants learning from LMIC surgeons [26, 27]. This approach is endorsed by the United States National Institute of Health and exemplified by Stawicki et al., who presented a case demonstrating the exchange of operative experience and mentorship to meet accreditation requirements while incorporating specific educational and competency-based objectives for both parties [28]. This mentorship enables trainees from HICs to acquire surgical and research skills relevant to resource-constrained settings, which they may not have exposure to in their HIC environment [6]. For instance, trainees in the United States are increasingly performing minimal-access surgery, resulting in less experience with open surgery. They would benefit from rotations in LMIC institutions that provide valuable exposure to open surgery and surgical practices in resource-limited settings [11, 29]. While the perception of LMIC surgeons towards the motivations of HIC trainees for their rotations in LMIC institutions remains uncertain, our study unveils that a significant 82% of LMIC surgeons are inclined to provide training opportunities to medical students, surgical trainees, and residents from HIC institutions. As more programs consider adopting this approach, there could be a need for accrediting bodies like the Accreditation Council for Graduate Medical Education and the Liaison Committee on Medical Education to develop guidelines that help promote fairness in this process for accredited medical programs.

### Limitations

Certain limitations are present in this study with regards to our chosen methodology and the responses we acquired. Firstly, the study survey was solely available in English, potentially leading to limited participation from non-English speaking countries and individuals and possible unintended survey response biases among individuals with limited English proficiency. We relied on social media and international collaborators for our survey distribution, restricting our survey’s reach to only areas where we had collaborators. Even within countries where collaboration was established, there could be diverse opinions, and the quantity of responses collected may not comprehensively reflect the perspectives of academic surgeons within those specific settings. To further delve into the underlying reasons for the viewpoints held by LMIC academic surgeons, forthcoming research should consider integrating interviews and other qualitative methods. These interviews can focus on investigating collaborations between HICs and LMICs, with particular emphasis on bidirectional training. Subsequent studies should also delve into potential remedies for additional factors that contribute to why LMIC academic surgeons attach greater importance to collaborations with HICs as opposed to independent endeavors.

## Conclusions

Although a significant number of LMIC surgeons demonstrate a readiness to participate in academic global surgery, obstacles remain in effectively translating their research achievements into avenues for advancing their careers within their institutions and increasing representation of their research in global surgery discourse. This study highlighted the presence of these challenges, encompassing aspects such as the availability of national research funding for academic global surgery (AGS), which we observed to correspond with the income level of these nations. Additionally, concerns encompass limited institutional funding and an absence of clear pathways for academic progress through AGS research. Consequently, LMIC surgeons tend to lean towards partnering with HICs to mitigate the effects of these hindrances. We hope that the global surgery community will actively address these barriers, fostering sustainable growth in the influence of LMIC representation and contributions. This, in turn, should reduce dependence on collaboration with HICs and elevate the significance of autonomous contributions to academic global surgery by frontline practitioners in LMICs.

## Supporting information

S1 Checklist Inclusivity in global research questionnaire. (DOCX)

S1 Text Collaborative authorship. (DOCX)

S1 Data Survey dataset. (CSV)
