# Supplementary material for: Beyond the Ivory Tower: Perception of academic global surgery by surgeons in low- and middle-income countries
Source: PLOS Glob Public Health. 2024 Mar 14;4(3):e0002979. doi: 10.1371/journal.pgph.0002979 (PMC10939292; doi:10.1371/journal.pgph.0002979)
Supplement: S1 Text — (DOCX) [file pgph.0002979.s002.docx]

**Collaborative Authorship**

**Steering Committee:** Arinzechukwu Nwagbata (co-first author)*, Rohini Dutta (co-first author)*, Samarvir Jain, Neil Thivalapill, Anusha Jayaram, Isabella Faria, Anita Gadgil, Nobhojit Roy, Nakul Raykar

**Protocol and Survey Design Team:** Rohini Dutta, Arinzechukwu Nwagbata, Anusha Jayaram, Samarvir Jain, Anna Alaska Pendleton, Soham Bandyopadhyay, Mandeep Grewal, Isabella Faria, Russell Seth Martins, Geetu Bhandoria, Anurag Mishra, Shilpa Khanna, Monali Mohan, Anita Gadgil, Nobhojit Roy, Nakul Raykar

**Data Collection Team:** Samarvir Jain, Isabella Faria, Anshul Mahajan, Rohini Dutta, Arinzechukwu Nwagbata, Vignesh Veerappan, Tarinee Kucchal, Juan Carlos Puyana, Geetu Bhandoria, Anita Gadgil, Nobhojit Roy, Nakul Raykar

**Data Analysis Team:** Neil Thivalapill, Arinzechukwu Nwagbata, Anusha Jayaram, Isaac Alty, Nakul Raykar

**Writing Team:** Arinzechukwu Nwagbata, Rohini Dutta, Anusha Jayaram, Isabella Faria, Isaac Alty, Anita Gadgil, Nobhojit Roy, Nakul Raykar

**Country Leads: Afghanistan:** Ahmad Neyazi; **Algeria:** Anisse Tidjane, Salah Eddine Oussama Kacimi; **Argentina:** Ayla Gerk Rangel; **Bangladesh:** Tasnim Shahriar; **Benin:** Mystère Djenontin-Agossou; **Bulgaria:** Krassimira Georgieva Zaykova; **Burkina Faso:** Abdoul Razak Sakande Zongo; **Burundi:** Arnaud Iradukunda, Marorerwa Audry; **Cabo Verde:** Andrea Rosienne Monteiro Nascimento; **China:** Lanhui Huang; **Colombia:** Marianna Castellanos **Commonwealth of Dominica:** Gloria Etim; **Costa Rica:** Adriana Montalvan Guasch, Nicole Montagne Bonilla; **Côte d’Ivoire:** Yaswa Djanwet; **Dominican Republic:** Nicolas Guzman; **Ecuador:** Juan Figueroa; **Egypt:** Dareen Abdallah; **El Salvador:** Manuel Alejandro Orellana Olmedo; **Ethiopia:** Aemon Berhane Fissha; **Fiji:** Joji Lesi; **Gambia:** Yankuba Jabbie; **Ghana:** Ama Ohene-Asi Dokyi, Christian Akore Agyeman; **Grenada:** Charlotte Gonikman; **Guatemala:** Juan Manuel Billeb Saca; **Guyana:** Muoneke Chidera Kingsley; **Haïti:** Tayana Jean Pierre; **Honduras:** Iving Elena Alvarado Carías;   **India:** Dhruv Nath Ghosh, Malika Gupta, Parvez David Haque, Anshul Mahajan; **Indonesia:** Jason Phowira; **Iran:** Negin Jarrah; **Iraq:** Saher Fuad, May Saad Al-jorani**; Kenya:** Emma Nalianya; **Kyrgyzstan:** Tooba Khursheed; **Lesotho:** Nthabiseng Tsoeu; **Libya:** Ekram Omar, Sharashi; **Malaysia:** Marjorie Ong Jia Yi; **Maldives:** Reem Zahir; **Mauritius:** Siddhant Arora; **Mexico:** Laura Patricia Aguilar Franco, Alejandro Zavala Contreras; **Mongolia:** Khatanzaya Sukhgerel; **Morocco:** Hajar Essangri, Moniba Korch, Amine Souadka, **Namibia:** Rashid Pueya Nashidengo; **Nigeria:** Adaolisa EbelechukwuNwagbata; **Pakistan:** Russell Seth Martins; **Paraguay:** Isabella dos Santos Pereira; **Philippines:** Terence M. Lapeñas; **Russia:** Brenda Gouvea Feres; **Rwanda:** Jean D’Amour Niyonkuru; **Serbia:** Šekler Stefan; **Sierra Leone:** Abdul Karim Bah; **Sri Lanka:** Navaneeth Krishna; **Sudan:** Mohammed Adil Elhussein Hajhamed; **Syria:** Jezel Albahry; **Tanzania:** Winfrida R. Burchards; **Thailand:** Arnav Mahajan, Thawin Techapongsatorn; **Uganda:** Wamala Nicholas Kisaakye; **Ukraine:** Alagbo Habib Olatunji; **Sierra Leone:** Mohamed Jambai Kanneh; **Uzbekistan:** Kamila Narkulova; **Vanuatu:** Ailson Nango Stanley; **Vietnam:** Hoang Pham Nguyen Tuyen; **Zimbabwe:** Shelton Choeni
